# Revisiting Wernicke’s sejunction theory: toward a mechanistic understanding of mental illness

**DOI:** 10.1192/j.eurpsy.2026.11800

**Published:** 2026-07-31

**Authors:** I. I. Grechenliev, S. R. Stoyneva

**Affiliations:** 1 University Hospital for Neurology and Psychiatry “Sveti Naum”; 2 Psychiatry Clinic, UMHAT “Aleksandrovska”, Sofia, Bulgaria

## Abstract

**Introduction:**

In most medical fields, disease entities are perceived as dysfunctions in particular systems. This entails a mechanistic view of pathology rooted in a solid physiological framework. The biomedical paradigm in psychiatry, however, has seldom provided such rigorous explanatory capabilities with regard to mental illnesses. Psychopathology is often conceived in a merely descriptive manner, while psychophysiology has been heavily underdeveloped or neglected throughout most of the field’s existence.

Since Carl Wernicke’s introduction of the idea of functional disconnections in the brain as causes for mental illness, his so-called “theory of sejunction”, few of the many subsequent psychiatric “traditions” have provided a more coherent, exhaustive and objectifiable explanatory model for psychiatric disorders. However, advances in computational psychiatry, connectomics and theoretical neurobiology, have once more revitalized the disconnection hypothesis for some of the most severe psychiatric ailments.

**Objectives:**

The aim of the current presentation is to emphasize the importance of adopting a mechanistic view of psychopathology in the hope that future developments in psychiatric diagnosis and treatment will utilize a theoretically sound framework.

**Methods:**

To accomplish the stated objective, some preliminary psychophysiological foundations, extending from Wernicke’s original work, will be presented. Namely, the three domains of functional associations in the brain (psychosensory, intrapsychic and psychomotor), as well as three control systems (affectivity, logical capacities and volition) will serve a pivotal role in the proper conceptualization of abnormal mental states.

**Results:**

Ultimately, specific psychopathological phenomena (e.g. sensory misperceptions, catatonic symptoms, formal thought disorders, etc.) prove capable of being reframed in terms of plausible control system dysfunctions in particular domains. Thus, the association between specific disruptions in brain functioning and observed pathological behaviors is established.

**Conclusions:**

Through the cogent construction of a neurofunctionally grounded theory of psychopathology, where each abnormal mental function may be logically inferred from its mechanisms of development, a potential alternative to current nosological paradigms has been provided.

**Disclosure of Interest:**

None Declared

